# Modeling Invasive Aspergillosis: How Close Are Predicted Antifungal Targets?

**DOI:** 10.3390/jof6040198

**Published:** 2020-09-30

**Authors:** Thomas J. Walsh, Ruta Petraitiene, Vidmantas Petraitis

**Affiliations:** 1 Department of Medicine, Division of Infectious Diseases, Transplantation-Oncology Infectious Diseases Program, Weill Cornell Medicine of Cornell University, New York, NY 10021, USA; rop2016@med.cornell.edu (R.P.); vip2007@med.cornell.edu (V.P.); 2 Department of Pediatrics, Weill Cornell Medicine of Cornell University, New York, NY 10021, USA; 3 Department of Microbiology & Immunology, Weill Cornell Medicine of Cornell University, New York, NY 10021, USA

**Keywords:** aspergillosis, animal models, antifungal agents, translational medicine

## Abstract

Animal model systems are a critical component of the process of discovery and development of new antifungal agents for treatment and prevention of invasive aspergillosis. The persistently neutropenic rabbit model of invasive pulmonary aspergillosis (IPA) has been a highly predictive system in identifying new antifungal agents for treatment and prevention of this frequently lethal infection. Since its initial development, the persistently neutropenic rabbit model of IPA has established a strong preclinical foundation for dosages, drug disposition, pharmacokinetics, safety, tolerability, and efficacy for deoxycholate amphotericin B, liposomal amphotericin B, amphotericin B lipid complex, amphotericin B colloidal dispersion, caspofungin, micafungin, anidulafungin, voriconazole, posaconazole, isavuconazole, and ibrexafungerp in treatment of patients with invasive aspergillosis. The findings of combination therapy with a mould-active triazole and an echinocandin in this rabbit model also predicted the outcome of the clinical trial for voriconazole plus anidulafungin for treatment of IPA. The plasma pharmacokinetic parameters and tissue disposition for most antifungal agents approximate those of humans in persistently neutropenic rabbits. Safety, particularly nephrotoxicity, has also been highly predictive in the rabbit model, as exemplified by the differential glomerular filtration rates observed in animals treated with deoxycholate amphotericin B, liposomal amphotericin B, amphotericin B lipid complex, and amphotericin B colloidal dispersion. A panel of validated outcome variables measures therapeutic outcome in the rabbit model: residual fungal burden, markers of organism-mediated pulmonary injury (lung weights and infarct scores), survival, and serum biomarkers. In selected antifungal studies, thoracic computerized tomography (CT) is also used with diagnostic imaging algorithms to measure therapeutic response of pulmonary infiltrates, which exhibit characteristic radiographic patterns, including nodules and halo signs. Further strengthening the predictive properties of the model, therapeutic response to successfully developed antifungal agents for treatment of IPA has been demonstrated over the past two decades by biomarkers of serum galactomannan and (1→3)-β-D-glucan with patterns of resolution, that closely mirror those documented responses in patients with IPA. The decision to move from laboratory to clinical trials should be predicated upon a portfolio of complementary and mutually validating preclinical laboratory animal models studies. Other model systems, including those in mice, rats, and guinea pigs, are also valuable tools in developing clinical protocols. Meticulous preclinical investigation of a candidate antifungal compound in a robust series of complementary laboratory animal models will optimize study design, de-risk clinical trials, and ensure tangible benefit to our most vulnerable immunocompromised patients with invasive aspergillosis.

## 1. Introduction

Animal model systems are a critical component of the process of discovery in development of new antifungal agents for treatment and prevention of acute invasive aspergillosis. Model systems of invasive pulmonary aspergillosis have been developed and studied in mice, rats, guinea pigs, and rabbits for development of new antifungal agents [1,2,3,4,5]. The purpose of this paper will be to review the markers of therapeutic response used in these systems and their predictability for clinical trials. This paper will particularly focus on the contributions and lessons learned from the rabbit model system of invasive pulmonary aspergillosis.

The objectives of this review are (1) to review the markers used in laboratory systems in development of new antifungal agents, (2) to assess the predictability of these markers for predicting outcome in clinical trials, and (3) to identify unmet needs and new directions for markers in preclinical and clinical trials. Given the broad scope of this important topic, we will focus especially on the advances in these areas accomplished through the persistently neutropenic and immunocompromised rabbit models of invasive pulmonary aspergillosis.

## 2. Rabbit Models of Invasive Pulmonary Aspergillosis

### 2.1. Translational Research Model

**Figure 1:**
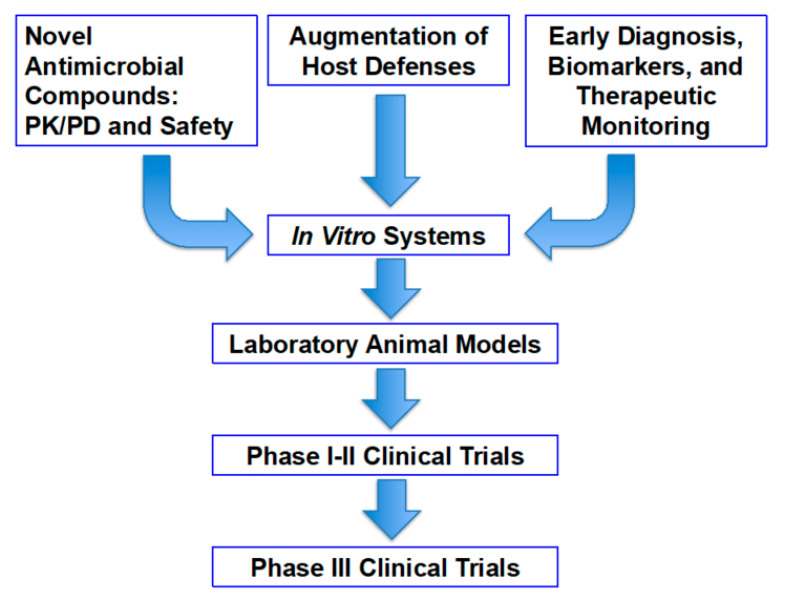
The strategies for translational research are achieved through a three-pronged approach that constitutes the pillars of management of immunocompromised patients.

Within the Infectious Diseases Translational Research Laboratory at Weill Cornell Medicine of Cornell University, we adapt a translation approach of observing the problems of invasive fungal infections and other life-threatening infectious diseases at the bedside and returning to the laboratory to work in collaboration, within the institution, as well as with partners in universities, government, industry, and foundations to address the challenges of invasive fungal infections. The strategies for translational research are achieved through a three-pronged approach that constitutes the pillars of management of immunocompromised patients (Figure 1).

The first pillar is that of development of treating the infection with a novel antimicrobial compound and further investigating its pharmacokinetics and pharmacodynamics as well as safety and tolerability. The second pillar of investigation is to develop new approaches toward augmentation of host defense or amelioration of immunosuppression. Finally, the third pillar of this translational research strategy is to develop biomarkers for early diagnosis and therapeutic monitoring of antifungal response. These strategies are employed through a series of in vitro systems, clinically predictive laboratory animal models, and implementation of phase 1 and phase 2 clinical trials. Following an assessment of the data of safety, tolerability, and efficacy from phase 1 and phase 2 clinical trials, phase 3 trials may then be developed.

### 2.2. Methodological Establishment of the Persistently Neutropenic Rabbit Model of Invasive Pulmonary Aspergillosis

**Figure 2:**
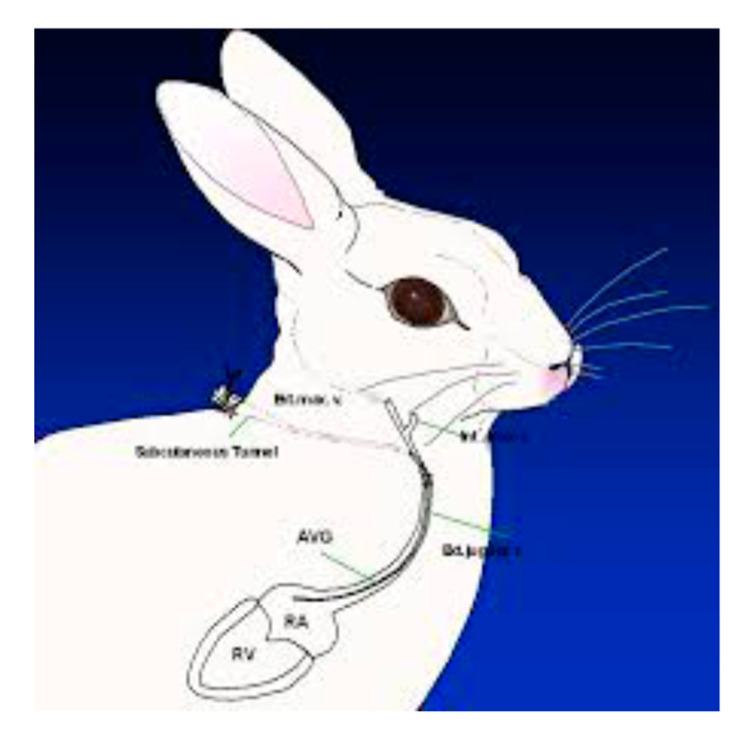
New Zealand white rabbit with tunneled central Silastic venous catheter. The vascular catheter is inserted into the right external jugular vein and then tunneled subcutaneously to exit in the midline anteriorly to the scapulae.

The persistently neutropenic rabbit model of invasive pulmonary aspergillosis has been a highly predictive system in identifying new antifungal agents for treatment and prevention of this frequently lethal disease. Paramount to the success of the persistently neutropenic rabbit model of invasive pulmonary aspergillosis is the presence of the central Silastic venous catheter, which permits atraumatic venous access [6]. The vascular catheter allows for the administration of multiple agents including immunosuppressive and cytotoxic chemotherapeutic agents, antimicrobial agents for prevention of superimposed bacterial infections, and investigational agents administered by parenteral (IV) route of administration (Figure 2). The vascular catheter also permits repeated venous access for pharmacokinetic sampling, toxicity sampling, and serial monitoring of biomarkers.

Persistent neutropenia is induced by administration of cytosine arabinoside (Ara-C) [7]. Doses are adjusted for both induction and maintenance of profound and persistent neutropenia, similar to that which is achieved in treatment of hematological malignancies, particularly acute myelogenous leukemia. Corticosteroids (methylprednisolone) are also administered to further impair pulmonary alveolar macrophage function. Further immune modulation can be obtained by administration of cyclosporine. This induction of profound and persistent neutropenia requires intensive support of care with empirical antibacterial therapy for prevention of life-threatening bacterial infections and close at least twice daily monitoring as well 24/7 on-call schedule.

### 2.3. Pathophysiology of Experimental Invasive Pulmonary Aspergillosis

**Figure 3:**
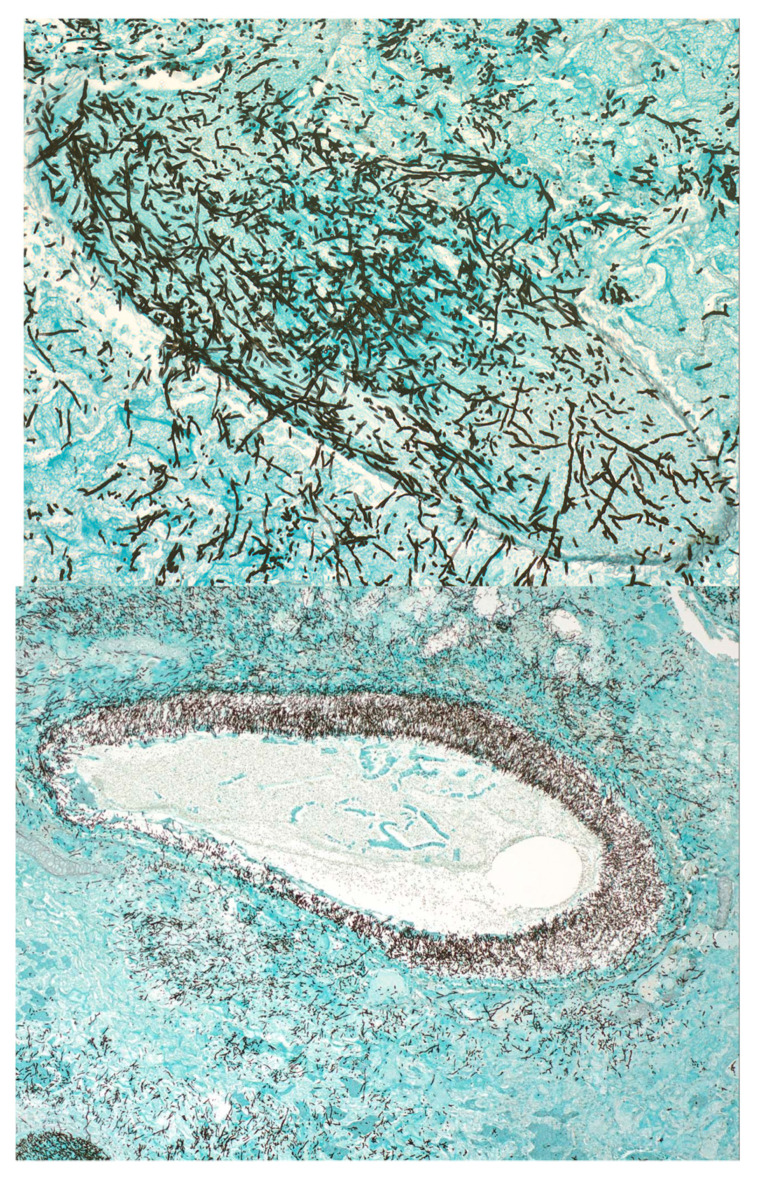
Histopathology of experimental pulmonary aspergillosis in the persistently neutropenic rabbit model depicts dichotomously branching septated hyphae of *Aspergillus fumigatus* invading blood vessels with thrombosis and extension into adjacent alveolar tissue (Grocott-Gomori’s methenamine silver (GMS) stain; original magnifications: upper panel (200×); lower panel (40×)).

**Figure 4:**
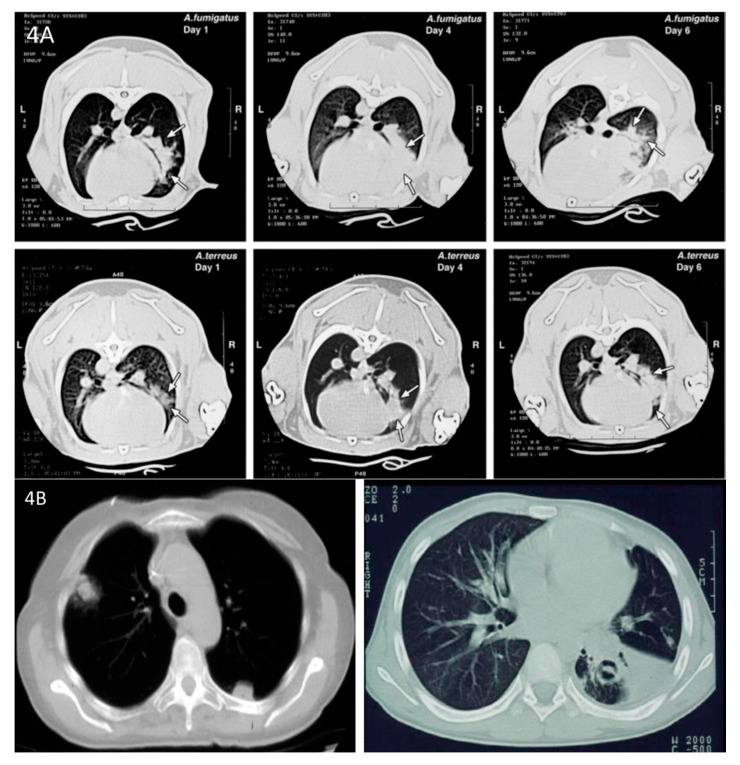
(**A**)**.** Computed tomography (CT) scan of the rabbit thorax with invasive pulmonary aspergillosis demonstrates segmental and nodular infiltrates with halo signs on days 1, 4, and 6 post inoculation. (**B**). Chest CT scans of patient with invasive pulmonary aspergillosis caused by *A. fumigatus* reveals nodular infiltrates with halo sign (left panel) and wedge-shaped segmental infiltrate with crescent sign (right panel).

The rabbit model of invasive pulmonary aspergillosis closely simulates the pathogenesis of that which is seen in immunocompromised patients, particularly those with hematological malignancies and hematopoietic stem cell transplantation [8]. An accurately calibrated and quantified inoculum of *Aspergillus* conidia is administered endotracheally while rabbits are under general anesthesia. This allows for carefully quantified colonization of the tracheobronchial tree by *Aspergillus fumigatus* or other *Aspergillus* spp. as similar to that, which occurs in immunocompromised patients; i.e., as immune suppression continues, colonization of the tracheobronchial tree progresses with angioinvasion and hemorrhagic infarction, resulting in a nodular segmental pneumonia (Figure 3). The pneumonic infiltrates have been well characterized by serial CT scans, where one is able to readily observe the development of halo sign and wedge-shaped infiltrates that are characteristic of the lesions of human invasive pulmonary aspergillosis (Figure 4) [9]. Therapy is initiated twenty-four hours after inoculation, when pulmonary infiltrates are established. This initiation therefore reflects the clinical features of development of pulmonary infiltrates by CT scan as the basis for initiation of antifungal therapy. Investigational and standard control antifungal agents are administered for 12–14 days.

### 2.4. Clinical Impact of the Rabbit Models of Experimental Invasive Pulmonary Aspergillosis

Since its initial development, the persistently neutropenic rabbit model of invasive pulmonary *Aspergillosis* has established a strong preclinical foundation for dosage, drug disposition, pharmacokinetics, safety, tolerability, and efficacy of new antifungal agents [10]. The rabbit model system laid critical preclinical foundations for the study of 14 antifungal agents that were studied in clinical trials (Table 1). These include five formulations of polyenes, three echinocandins, five antifungal triazoles, and ibrexafungerp (first-in-class triterpene inhibitors of (1→3)-β-D-glucan synthase).

## 3. Lipid Formulations of Amphotericin B

The rabbit model system for invasive pulmonary aspergillosis in the persistently neutropenic host was initially studied in examining the efficacy of unilamellar liposomal amphotericin B (AmBisome) in treatment of pulmonary aspergillosis in persistently neutropenic rabbits [7]. Although earlier work had demonstrated the sensitivity of galactomannan in detection of experimental disseminated aspergillosis, this study, to our knowledge, also incorporated the first documented use of serum galactomannan as a biomarker for infection of experimental invasive pulmonary *Aspergillosis*, and also was the first to examine the sensitivity in detection of this disease. Francis et al. also examined another biomarker of D-mannitol in the detection of invasive pulmonary aspergillosis in bronchoalveolar lavage (BAL) fluid. This study examined a humanized dosing liposomal amphotericin B at 5 mg/kg per day vs. 1 mg/kg per day, wherein a liposomal formulation was found to be more effective and safer with using a series of outcome biomarkers demonstrating increasing survival, a reduction in the number of viable organisms (residual fungal burden), a decrease in organism-mediated pulmonary injury (lung weights and pulmonary infarct score), and a reduction in nephrotoxicity, as measured by serum creatinine.

The efficacy of liposomal amphotericin B in primary treatment of invasive pulmonary aspergillosis was established in a randomized trial comparing a high loading dose regimen with a standard dose (AmBiLoad trial) [70]. In this double-blind trial, patients with proven or probable invasive aspergillosis or other mold infection were randomized to receive liposomal amphotericin B at either 3 or 10 mg/kg per day for fourteen days, followed by 3 mg/kg per day. The overall survival rate at 12 weeks of liposomal amphotericin B at 3 mg/kg was 72% and the overall response rate at 12 weeks was 50%. Liposomal amphotericin B was also studied in a randomized double-blind trial studying the safety and efficacy of liposomal amphotericin B vs. conventional deoxycholate amphotericin B for empirical antifungal therapy in persistently febrile neutropenic patients.

In further study of the predictive value of the rabbit model system of acute invasive pulmonary aspergillosis for human antifungal therapeutics, we investigated the comparative pharmacodynamics of amphotericin B deoxycholate, amphotericin B lipid complex (ABLC), and liposomal amphotericin B (LAMB) against invasive pulmonary aspergillosis [11]. The study demonstrated that the near maximal antifungal activity by measurement of galactomannan and (1→3)-β-D-glucan showed near maximal antifungal activity of deoxycholate amphotericin B at 1 mg/kg per day and the lipid formulations of amphotericin B, ABLC, LAMB, at 3–5 mg/kg per day. This study further demonstrated that all formulations of amphotericin B induced a dose-dependent reduction in markers of pulmonary injury as well as circulating fungus related biomarkers. A clinical dosage of LAMB at 3 mg/kg per day was successfully predicted to cause complete suppression of serum galactomannan and serum (1→3)-β-D-glucan levels in the majority of patients. Resolution of serum galactomannan, (1→3)-β-D-glucan, and proteomic biomarkers has been consistently correlated with successful outcome since the original observations of serum galactomannan in the rabbit model of experimental pulmonary aspergillosis [7,71,72,73,74].

## 4. Antifungal Triazoles

The rabbit model of invasive pulmonary aspergillosis has further been studied in the investigation of antifungal triazoles. Among the major advances in the development of anti-mould triazoles are itraconazole, posaconazole, voriconazole, and isavuconazole.

Posaconazole was studied in the persistently neutropenic rabbit model of invasive aspergillosis for the study of treatment and prevention of invasive aspergillosis [5,12,75]. Posaconazole was studied at 2, 6, and 20 mg/kg of body weight PO in comparison to that of itraconazole at 2, 6, and 20 mg/kg PO and in comparison to deoxycholate amphotericin B at 1 mg/kg IV. Posaconazole conferred a significant reduction in residual fungal burden, mean pulmonary infarct score, and mean total lung weight at 6 and 20 mg/kg per day. By comparison, itraconazole had only minimal effect on these parameters despite similar plasma concentrations. The efficacy of posaconazole appeared to be numerically greater in reducing pulmonary infarct score and mean pulmonary residual fungal burden (log CFU/g) but did not achieve statistical significance. In aggregate, the animals treated with posaconazole or deoxycholate amphotericin B had significantly greater survival than those untreated controls or those treated with itraconazole. Corresponding to this antifungal activity, posaconazole achieved significant reduction in serum galactomannan at 6 and 20 mg/kg per day and in serum (1→3)-β-D-glucan also at 6 and 20 mg/kg per day. Only itraconazole at 20 mg/kg per day but not at 2 and 6 mg/kg per day achieved comparable reductions in serum galactomannan. The plasma concentrations of posaconazole and itraconazole achieved at 2, 6, and 20 mg/kg per day were relatively similar in the different dosage regiments at 2 mg/kg per day but differed with slightly higher concentrations at 6 and 20 mg/kg per day. Moreover, when the studied drugs were administered for prevention of invasive pulmonary aspergillosis, posaconazole was also superior to itraconazole for the prevention of invasive pulmonary aspergillosis in the persistently neutropenic rabbit model. These findings laid the preclinical foundation for the open-label salvage study of posaconazole for treatment of invasive pulmonary aspergillosis in patients who were refractory to or intolerant of conventional therapy [63] as well as for establishing the scientific preclinical foundation for a randomized control trial of posaconazole for prevention of invasive fungal infections in patients with hematological malignancies [64].

The clinical trial for treatment of invasive aspergillosis with posaconazole in patients who were refractory to or intolerant of conventional therapy studied posaconazole in oral suspension at 800 mg per day in divided doses as monotherapy [63]. In this open-label multicenter study, global response to salvage therapy found an overall success rate of 42% for 107 posaconazole recipients in comparison to 26% of externally controlled subjects (odds ratio 4.06 (95% confidence intervals 1.50–11.04), *P* =0.006). Among the control group, there were 80 patients treated with amphotericin B, 49 with itraconazole, 45 with amphotericin B and itraconazole, and other therapies with 36. The survival distribution in the two groups revealed a significant difference (*P* = 0.2003). The overall response correlated with the mean plasma concentration of posaconazole. Patients with a mean plasma C_max_ of 1480 ng/mL and C_average_ of 1250 ng/mL (*n* = 12) had a 75% response rate in comparison to other patients at ≤852 mean plasma C_max_ concentration and ≤ 715 mean average concentration, where response was ≤53% at or below these concentrations. These pharmacodynamic findings correlated strongly with the concentration thresholds observed in the preclinical neutropenic rabbit models of invasive pulmonary aspergillosis.

The rabbit model system also laid an experimental preclinical foundation for and predicted the clinical outcome of the prophylaxis of the study of posaconazole vs. fluconazole or itraconazole for prophylaxis in patients with neutropenia [64]. This study demonstrated that in patients, undergoing chemotherapy for acute myelogenous leukemia or myelodysplastic syndrome, posaconazole significantly reduced invasive fungal infections, including invasive aspergillosis, more effectively than fluconazole and itraconazole, and significantly improved overall survival.

## 5. Combination Antifungal Therapy

Three major preclinical studies in the persistently neutropenic rabbit model using the broad range of therapeutic response markers built the preclinical foundation for combination therapy with mould-active triazole plus an echinocandin for primary treatment of invasive aspergillosis. Based on the extensive in vitro observations that a combination of a mould-active triazole and an echinocandin were additive synergistic against *Aspergillus fumigatus*, we hypothesized that the simultaneous inhibition of ergosterol biosynthesis affecting the fungal the cell membrane and (1→3)-β-D-glucan production altering the cell wall would result in improved outcome in experimental invasive pulmonary aspergillosis. These studies established proof of concept that the combination of a mould-active triazole and an echinocandin would be active against invasive pulmonary aspergillosis in patients with this frequently lethal infection [34,35,42,43,51,52,53,54,55,60,76,77,78,79,80,81].

Consistent with these in vitro and in vivo studies, there is a strong correlation between concentration-dependent and dose-dependent interactions between anidulafungin and voriconazole by Bliss independence drug interaction analysis [42]. The in vitro observations demonstrated a dose-dependent interaction between anidulafungin and voriconazole by Bliss independence drug interaction analysis. The combination of voriconazole and anidulafungin in vivo was superior to either single agent in improving survival, decreasing pulmonary organism-mediated pulmonary injury as measured by pulmonary infarct score and diminishing lung weight as markers of pulmonary injury. Moreover, the combination of voriconazole and anidulafungin significantly reduced the residual fungal burden in comparison to single agents. These findings were consistent with prior studies of echinocandin and antifungal triazoles in vitro and in vivo. The additive synergistic interaction of anidulafungin plus voriconazole was further exemplified in measurement of the CT pulmonary infiltrate volume scores, a significant reduction in the combination compared to single agents alone and untreated control. Further demonstrating the combination of anidulafungin plus voriconazole, serum galactomannan was significantly reduced by the combination regiments compared to single agents alone, as were bronchoalveolar lavage galactomannan levels. A post hoc analysis of the clinical trial of this combination also demonstrated that six-week mortality was significantly lower in combination therapy than monotherapy (15.7% (17/108) vs. 27.3% (30/110): difference = 11.5% (95% CI−22.71 to −0.40), *P* = 0.037) [82].

### Ibrexafungerp Plus Mould-Active Triazole in Combination Therapy

Building upon the observations of additive to synergistic interactions between mould-active triazoles and echinocandins against *A. fumigatus*, we further hypothesized that the combination of ibrexafungerp, which inhibits (1→3)-β-D-glucan synthase, and a mould-active triazole (isavuconazole), would be similarly active in vitro and in vivo [65]. Ibrexafungerp is a triterpene-derived molecule that inhibits (1→3)-β-D-glucan synthase in *Candida* spp. and *Aspergillus* spp. In vitro data demonstrated an additive to synergistic interaction through plus surface analysis that was further evaluated *in vivo*. When the combination of ibrexafungerp and isavuconazole was studied in the persistently neutropenic rabbit model of invasive pulmonary aspergillosis, the combination therapy was significantly more active in all outcome parameters in comparison to that of the single agents. The findings of this observation have led to the development of a new phase 2B randomized control trial of ibrexafungerp plus voriconazole, as the mould-active triazole, vs. voriconazole alone, as standard of care (ClinicalTrials.gov Identifier: NCT03672292).

## 6. Olorofim

Olorofim, a first-in-class orotomide inhibitor of dihydroorotate dehydrogenase in the pyrimidine salvage pathway, was studied in the rabbit model of acute IPA [69]. Using serum galactomannan as the primary pharmacodynamic endpoint, the authors found that *C*_min_ and *C*_min_/MIC targets of 0.1 and 3.3, respectively, produced in vivo effects similar to those reported for isavuconazole administered in human exposures. In addition to serum galactomannan, survival, and histopathology were analyzed in the study. These studies contributed important data to determination of dosage regimens in ongoing clinical trials (ClinicalTrials.gov https://clinicaltrials.gov/ct2/show/NCT03583164; https://clinicaltrials.gov/ct2/show/NCT04039880).

### Chronic Invasive Pulmonary Aspergillosis in the Non-Neutropenic Rabbit Model

Many patients who develop invasive pulmonary aspergillosis are not neutropenic. Such patients include those receiving corticosteroid therapy, graft vs. host disease, autoimmune disease, solid organ transplantation, and cystic fibrosis. We have established a model of chronic invasive pulmonary aspergillosis, which recapitulates many of the histological, microbiological, and immunological characteristics observed in these vulnerable populations.

The pathophysiology of *Aspergillus* infection in the non-neutropenic rabbit model of chronic pulmonary aspergillosis differs markedly from that of the persistently neutropenic rabbit of acute invasive pulmonary aspergillosis [4,47,83]. Rabbits treated with cyclosporin A plus methylprednisolone develop a chronic pyogranulomatous inflammatory response with neutrophilic and monocytic infiltrates and inflammatory necrosis with 100% survival in contrast to the 100% mortality, angioinvasion and hemorrhagic infraction in the persistently neutropenic rabbit model. These immunophenotypic and histological features in the rabbit models correlate with those observed in patient populations with similar immunosuppressive therapies [84].

As an example of the applications of this system, itraconazole and amphotericin B were compared in this model system immunosuppressed by methylprednisolone and cyclosporine A. Both agents had in vivo antifungal activity: however, amphotericin B was more effective than itraconazole in reducing the residual fungal burden and pulmonary lesions. As the overall survival in this animal model over thirty days is virtually 100%, survival is not used a marker of response. In measuring response, however, to itraconazole, there was a strong inverse correlation between concentrations of itraconazole and the tissue burden of *Aspergillus fumigatus*. Indeed an inhibitory sigmoidal maximum effect model predicted a significant pharmacodynamic relationship between itraconazole concentrations in plasma and antifungal activity as measured by residual fungal burden of *Aspergillus fumigatus*. Moreover, the model recapitulated the human drug–drug interaction observed between cyclosporin A and itraconazole. The model system also reflected the greater nephrotoxicity of amphotericin B in comparison to that of itraconazole.

Expression of serum galactomannan and (1→3)-β-D-glucan was studied in serum and BAL fluid simultaneously in both models of acute invasive pulmonary aspergillosis in the persistently neutropenic rabbit model and in the chronic pulmonary aspergillosis model [4]. Specifically, these biomarkers were studied in response to the treatment by liposomal amphotericin B. The concentration–time curves for serum (1→3)-β-D-glucan and serum galactomannan index were significantly higher in the persistently neutropenic rabbit model than in the chronic pulmonary aspergillosis model. By comparison, the therapeutic responses were similar to each other in comparison to untreated controls in both models. Moreover, host differences did not affect the concentration of the galactomannan index or the level of (1→3)-β-D-glucan in BAL fluid. The relationships of the higher expression of (1→3)-β-D-glucan and serum galactomannan correlated with a greater degree of intact hyphae and extensive angioinvasion by infiltrating hyphal forms of *Aspergillus fumigatus*.

The study demonstrated the implementation of biomarkers from preclinical data into clinical endpoint response criteria, with particular emphasis upon the impact of the rabbit models of acute and chronic invasive pulmonary aspergillosis and integration of that data into dosage and study design for clinical trials of which these systems have been highly predictive for efficacy, pharmacokinetics, and safety.

## Figures and Tables

**Table 1:** Antifungal agents, preclinical studies in the persistently neutropenic and non-neutropenic rabbit model systems of invasive pulmonary *Aspergillosis*, and related clinical trials.

Antifungal Agent (References)	Markers of In Vivo Therapeutic Response							
	RFB	PH-Score	Lung-Wt	Survival	Serum GMI	Serum BDG	CTS Score	PCR
Deoxycholate amphotericin B (DAMB) [11,12,13,14,15,16]	**√**	**√**	**√**	**√**	**√**		**√**	**√**
Liposomal amphotericin B (LAMB) [4,7,11,17,18]	**√**	**√**	**√**	**√**	**√**	**√**	**√**	**√**
Amphotericin B lipid complex ABLC) [11,19,20]	**√**	**√**	**√**	**√**	**√**			
Amphotericin B colloidal dispersion (ABCD) [21,22,23,24,25]	**√**	**√**	**√**	**√**				
Liposomal nystatin [26,27]	**√**	**√**	**√**	**√**				
Caspofungin [13,28,29,30,31,32,33]	√	√	√	√	**√**			
Micafungin [34,35,36,37,38,39,40,41]	√	√	√	√	√	**√**		
Anidulafungin [42,43,44,45,46]	√	√	√	√			**√**	
Itraconazole [12,47,48,49,50]	√	√	√	√	√			
Voriconazole [42,51,52,53,54,55,56,57,58,59]	√	√	√	√	√	**√**	**√**	
Ravuconazole [17,34,60,61,62]	√	√	√	√	√	**√**	**√**	
Posaconazole [5,12,63,64]	√	√	√	√	√	**√**	**√**	
Isavuconazole [35,65,66,67,68]	√	√	√	√	√	**√**		
Ibrexafungerp [65]	√	√	√	√	√	**√**		
Olorofim (F901318) [69]	**√**			**√**	**√**			

RFB: residual fungal burden; PH: pulmonary hemorrhage; Wt: weight; GMI: galactomannan index; CTS: CT scan.

## References

[1] Maertens J.A., Raad I.I., Marr K.A., Patterson T.F., Kontoyiannis D.P., Cornely O.A., Bow E.J., Rahav G., Neofytos D., Aoun M. (2016). Isavuconazole versus voriconazole for primary treatment of invasive mould disease caused by *Aspergillus* and other filamentous fungi (SECURE): A phase 3, randomized-controlled, non-inferiority trial. Lancet.

[2] Lepak A.J., Marchillo K., Vanhecker J., Andes D.R. (2013). Posaconazole pharmacodynamic target determination against wild-type and Cyp51 mutant isolates of *Aspergillus fumigatus* in an in vivo model of invasive pulmonary aspergillosis. Antimicrob. Agents Chemother..

[3] Schmitt H., Edwards F., Andrade J., Niki Y., Armstrong D. (1992). Comparison of azoles against aspergilli in vitro and in an experimental model of pulmonary aspergillosis. Chemotherapy.

[4] Wiederhold N.P., Najvar L.K., Vallor A.C., Kirkpatrick W.R., Bocanegra R., Molina D., Olivo M., Graybill J.R., Patterson T.F. (2008). Assessment of serum (1->3)-β-D-glucan concentration as a measure of disease burden in a murine model of invasive pulmonary aspergillosis. Antimicrob. Agents Chemother..

[5] Petraitiene R., Petraitis V., Bacher J.D., Finkelman M.A., Walsh T.J. (2015). Effects of host response and antifungal therapy on serum and BAL levels of galactomannan and (1→3)-β-D-glucan in experimental invasive pulmonary aspergillosis. Med. Mycol..

[6] Petraitiene R., Petraitis V., Groll A.H., Sein T., Piscitelli S., Candelario M., Field-Ridley A., Avila N., Bacher J., Walsh T.J. (2001). Antifungal activity and pharmacokinetics of posaconazole (SCH 56592) in treatment and prevention of experimental invasive pulmonary aspergillosis: Correlation with galactomannan antigenemia. Antimicrob. Agents Chemother..

[7] Walsh T.J., Bacher J., Pizzo P.A. (1988). Chronic Silastic central venous catheterization for induction, maintenance and support of persistent granulocytopenia in rabbits. Lab. Anim. Sci..

[8] Francis P.R., Lee J.W., Hoffman A.G.D., Peter J., Francesconi A., Bacher J., Shelhamer J., Pizzo P.A., Walsh T.J. (1994). Efficacy of unilamellar liposomal amphotericin B in treatment of pulmonary aspergillosis in persistently granulocytopenic rabbits: The potential role of bronchoalveolar D-mannitol and serum galactomannan as markers of infection. J. Infect. Dis..

[9] Hope W., Kruhlak M.J., Lyman C.A., Petraitiene R., Petraitis V., Francesconi A., Kasai M., Mickiene D., Sein T., Peter J. (2007). Pathogenesis of *Aspergillus fumigatus* and the kinetics of galactomannan in an in vitro model of early invasive pulmonary aspergillosis: Implications for antifungal therapy. J. Infect. Dis..

[10] Walsh T.J., Garrett K., Feurerstein E., Girton M., Allende M., Bacher J., Francesconi A., Schäufele R., Pizzo P.A. (1995). Therapeutic monitoring of experimental invasive pulmonary aspergillosis by ultrafast computerized tomography, a novel, noninvasive method for measuring responses to antifungal therapy. Antimicrob. Agents Chemother..

[11] Cornely O.A., Maertens J., Bresnik M., Ebrahimi R., Ullmann A.J., Bouza E., Heussel C.P., Lortholary O., Rieger C., Boehme A. (2007). Liposomal amphotericin B as initial therapy for invasive mold infection: A randomized trial comparing a high-loading dose regimen with standard dosing (AmBiLoad Trial). Clin. Infect. Dis..

[12] O’Sullivan C.E., Kasai M., Francesconi A., Petraitis V., Petraitiene R., Kelaher A.M., Sarafandi A.A., Walsh T.J. (2003). Development and validation of a quantitative real-time PCR assay using fluorescence resonance energy transfer technology for detection of *Aspergillus fumigatus* in experimental invasive pulmonary aspergillosis. J. Clin. Microbiol..

[13] Stergiopoulou T., Meletiadis J., Roilides E., Kleiner D.E., Schaufele R., Roden M., Harrington S., Dad L., Segal B., Walsh T.J. (2007). Host-dependent patterns of tissue injury in invasive pulmonary aspergillosis. Am. J. Clin. Pathol..

[14] Petraitiene R., Petraitis V., Groll A.H., Sein T., Schaufele R.L., Francesconi A., Bacher J., Avila N.A., Walsh T.J. (2002). Antifungal efficacy of caspofungin (MK-0991) in experimental pulmonary aspergillosis in persistently neutropenic rabbits: Pharmacokinetics, drug disposition, and relationship to galactomannan antigenemia. Antimicrob. Agents Chemother..

[15] Petraitis V., Petraitiene R., Lin P., Calis K., Kelaher A.M., Muray H.A., Mya-San C., Mickiene D., Bacher J., Walsh T.J. (2005). Efficacy and safety of generic amphotericin B in experimental pulmonary aspergillosis. Antimicrob. Agents Chemother..

[16] Petraitis V., Petraitiene R., Solomon J., Kelaher A.M., Murray H.A., Mya-San C., Bhandary A.K., Sein T., Avila N.A., Basevicius A. (2006). Multidimensional volumetric imaging of pulmonary infiltrates for measuring therapeutic response to antifungal therapy in experimental invasive pulmonary aspergillosis. Antimicrob. Agents Chemother..

[17] Hope W., Petraitis V., Petraitiene R., Aghamolla T., Bacher J., Walsh T.J. (2010). The initial 96 hours of invasive pulmonary aspergillosis: Histopathology, comparative kinetics of galactomannan and (1→3)-β-D-glucan, and consequences of delayed antifungal therapy. Antimicrob. Agents Chemother..

[18] Meletiadis J., Petraitis V., Petraitiene R., Lin P., Stergiopoulou T., Sein T., Bacher J., Kelaher A.M., Schaufele R.L., Walsh T.J. (2006). Triazole? Polyene antagonism in experimental invasive pulmonary aspergillosis: In vitro and in vivo correlation. J. Infect. Dis..

[19] Leenders A.C.A.P., Daenen S., Jansen R.L.H., Hop W.C.J., Löwenberg B., Wijermans P.W., Cornelissen J., Herbrecht R., Van Der Lelie H., Hoogsteden H.C. (1998). Liposomal amphotericin B compared with amphotericin B deoxycholate in the treatment of documented and suspected neutropenia-associated invasive fungal infections. Br. J. Haematol..

[20] Walsh T.J., Hiemenz J.W., Seibel N.L., Perfect J.R., Horwith G., Lee L., Silber J.L., Di Nubile M.J., Reboli A., Bow E. (1998). Amphotericin B lipid complex for invasive fungal infections: Analysis of safety and efficacy in 556 cases. Clin. Infect. Dis..

[21] Walsh T.J., Seibel N.L., Arndt C., Harris R.E., Di Nubile M.J., Reboli A., Hiemenz J., Chanock S.J. (1999). Amphotericin B lipid complex in pediatric patients with invasive fungal infections. Pediatr. Infect. Dis. J..

[22] Allende M.C., Lee J.W., Francis P., Garrett K., Dollenberg H., Berenguer J., Lyman C.A., Pizzo P.A., Walsh T.J. (1994). Dose-dependent antifungal activity and nephrotoxicity of amphotericin B colloidal dispersion in experimental pulmonary aspergillosis. Antimicrob. Agents Chemother..

[23] White M.H., Anaissie E.J., Kusne S., Wingard J.R., Hiemenz J.W., Cantor A., Gurwith M., Du Mond C., Mamelok R.D., Bowden R.A. (1997). Amphotericin B colloidal dispersion vs. amphotericin B as therapy for invasive aspergillosis. Clin. Infect. Dis..

[24] Anaissie E.J., Mattiuzzi G.N., Miller C.B., Noskin G.A., Gurwith M.J., Mamelok R.D., Pietrelli L.A. (1998). Treatment of invasive fungal infections in renally impaired patients with amphotericin B colloidal dispersion. Antimicrob. Agents Chemother..

[25] White M.H., Bowden R.A., Sandler E.S., Graham M.L., Noskin G.A., Wingard J.R., Goldman M., Van Burik J., McCabe A., Lin J. (1998). Randomized, double-blind clinical trial of amphotericin B colloidal dispersion vs. amphotericin B in the empirical treatment of fever and neutropenia. Clin. Infect. Dis..

[26] Bowden R., Chandrasekar P., Pietrelli L., Gurwith M., Safrin S., White M.H., Li X., Van Burik J.-A., Laverdiere M., Wingard J.R. (2002). A double-blind, randomized, controlled trial of amphotericin B colloidal dispersion versus amphotericin B for treatment of invasive aspergillosis in immunocompromised patients. Clin. Infect. Dis..

[27] Groll A.H., Gonzalez C.E., Giri N., Kligys K., Love W., Peter J., Feuerstein E., Bacher J., Piscitelli S.C., Walsh T.J. (1999). Liposomal nystatin against experimental pulmonary aspergillosis in persistently neutropenic rabbits: Efficacy, safety and non-compartmental pharmacokinetics. J. Antimicrob. Chemother..

[28] Offner F., Krcmery V., Boogaerts M., Doyen C., Engelhard D., Ribaud P., Cordonnier C., De Pauw B., Durrant S., Marie J.-P. (2004). Liposomal nystatin in patients with invasive aspergillosis refractory to or intolerant of amphotericin B. Antimicrob. Agents Chemother..

[29] Maertens J., Raad I., Petrikkos G., Boogaerts M., Selleslag D., Petersen F.B., Sable C.A., Kartsonis N., Ngai A., Taylor A. (2004). Efficacy and safety of caspofungin for treatment of invasive aspergillosis in patients refractory to or intolerant of conventional antifungal therapy. Clin. Infect. Dis..

[30] Aliff T.B., Maslak P., Jurcic J.G., Heaney M.L., Cathcart K., Sepkowitz K.A., Weiss M.A. (2003). Refractory *Aspergillus* pneumonia in patients with acute leukemia. Cancer.

[31] Maertens J., Glasmacher A., Herbrecht R., Thiebaut A., Cordonnier C., Segal B.H., Killar J., Taylor A., Kartsonis N., Patterson T.F. (2006). Multicenter, noncomparative study of caspofungin in combination with other antifungals as salvage therapy in adults with invasive aspergillosis. Cancer.

[32] Hiemenz J.W., Raad I.I., Maertens J.A., Hachem R.Y., Saah A.J., Sable C.A., Chodakewitz J.A., Severino M.E., Saddier P., Berman R.S. (2010). Efficacy of caspofungin as salvage therapy for invasive aspergillosis compared to standard therapy in a historical cohort. Eur. J. Clin. Microbiol. Infect. Dis..

[33] Walsh T.J., Teppler H., Donowitz G.R., Maertens J., Baden L.R., Dmoszyñska A., Cornely O.A., Bourque M.R., Lupinacci R.J., Sable C.A. (2004). Caspofungin versus liposomal amphotericin B for empirical antifungal therapy in patients with persistent fever and neutropenia. N. Engl. J. Med..

[34] Meletiadis J., Al-Saigh R.J., Velegraki A., Walsh T.J., Roilides E., Zerva L. (2011). Pharmacodynamic effects of simulated standard doses of antifungal drugs against *Aspergillus* species in a new in vitro pharmacokinetic/pharmacodynamic model. Antimicrob. Agents Chemother..

[35] Petraitis V., Petraitiene R., Hope W., Meletiadis J., Mickiene D., Hughes J.E., Cotton M.P., Stergiopoulou T., Kasai M., Francesconi A. (2009). Combination therapy in treatment of experimental pulmonary aspergillosis: In vitro and in vivo correlations of the concentration- and dose-dependent interactions between anidulafungin and voriconazole by bliss independence drug interaction analysis. Antimicrob. Agents Chemother..

[36] Walsh T.J., Adamson P.C., Seibel N.L., Flynn P.M., Neely M.N., Schwartz C., Shad A., Kaplan S.L., Roden M.M., Stone J.A. (2005). Pharmacokinetics, safety, and tolerability of caspofungin in children and adolescents. Antimicrob. Agents Chemother..

[37] Petraitis V., Petraitiene R., Groll A.H., Roussillon K., Hemmings M., Lyman C.A., Sein T., Bacher J., Bekersky I., Walsh T.J. (2002). Comparative antifungal activities and plasma pharmacokinetics of micafungin (FK463) against disseminated candidiasis and invasive pulmonary aspergillosis in persistently neutropenic rabbits. Antimicrob. Agents Chemother..

[38] Denning D.W., Marr K.A., Lau W.M., Facklam D.P., Ratanatharathorn V., Becker C., Ullmann A.J., Seibel N.L., Flynn P.M., Van Burik J.-A.H. (2006). Micafungin (FK463), alone or in combination with other systemic antifungal agents, for the treatment of acute invasive aspergillosis. J. Infect..

[39] Kontoyiannis D., Ratanatharathorn V., Young J.-A., Raymond J., Laverdière M., Denning D., Patterson T., Facklam D., Kovanda L., Arnold L. (2009). Micafungin alone or in combination with other systemic antifungal therapies in hematopoietic stem cell transplant recipients with invasive aspergillosis. Transpl. Infect. Dis..

[40] Van Burik J.-A.H., Ratanatharathorn V., Stepan D.E., Miller C.B., Lipton J.H., Vesole D.H., Bunin N., Wall D.A., Hiemenz J.W., Satoi Y. (2004). Micafungin versus fluconazole for prophylaxis against invasive fungal infections during neutropenia in patients undergoing hematopoietic stem cell transplantation. Clin. Infect. Dis..

[41] Santos R.P., Sánchez P.J., Mejias A., Benjamin D.K., Walsh T.J., Patel S., Jafri H.S. (2007). Successful medical treatment of cutaneous aspergillosis in a premature infant using liposomal amphotericin b, voriconazole and micafungin. Pediatr. Infect. Dis. J..

[42] Petraitis V., Petraitiene R., Sarafandi A.A., Kelaher A.M., Lyman C.A., Casler H.E., Sein T., Groll A.H., Bacher J., Avila N.A. (2003). Combination therapy in treatment of experimental pulmonary aspergillosis: Synergistic interaction between an antifungal triazole and an echinocandin. J. Infect. Dis..

[43] Meletiadis J., Stergiopoulou T., O’Shaughnessy E.M., Peter J., Walsh T.J. (2007). Concentration-dependent synergy and antagonism within a triple antifungal drug combination against *Aspergillus* species: Analysis by a new response surface model. Antimicrob. Agents Chemother..

[44] Seibel N.L., Schwartz C., Arrieta A., Flynn P., Shad A., Albano E., Keirns J., Lau W.M., Facklam D.P., Buell D.N. (2005). Safety, tolerability, and pharmacokinetics of micafungin (FK463) in febrile neutropenic pediatric patients. Antimicrob. Agents Chemother..

[45] Petraitis V., Petraitiene R., Groll A.H., Bell A., Callender D.P., Sein T., Schaufele R.L., McMillian C.L., Bacher J., Walsh T.J. (1998). Antifungal efficacy, safety, and single-dose pharmacokinetics of LY303366, a novel echinocandin B, in experimental pulmonary aspergillosis in persistently neutropenic rabbits. Antimicrob. Agents Chemother..

[46] Groll A.H., Mickiene D., Petraitiene R., Petraitis V., Lyman C.A., Bacher J.S., Piscitelli S.C., Walsh T.J. (2001). Pharmacokinetic and pharmacodynamic modeling of anidulafungin (LY303366): Reappraisal of its efficacy in neutropenic animal models of opportunistic mycoses using optimal plasma sampling. Antimicrob. Agents Chemother..

[47] Hope W., McEntee L., Livermore J., Whalley S., Johnson A., Farrington N., Kolamunnage-Dona R., Schwartz J., Kennedy A., Law D. (2017). Pharmacodynamics of the orotomides against *Aspergillus fumigatus*: New opportunities for treatment of multidrug-resistant fungal disease. mBio.

[48] Lee D.-G., Lee H., Yan J.L., Lin S.S., Aram J.A. (2019). Efficacy and safety of combination antifungal therapy in Korean haematological patients with invasive aspergillosis. Mycoses.

[49] Denning D.W., Lee J.Y., Hostetler J.S., Pappas P., Kauffman C.A., Dewsnup D.H., Galgiani J.N., Graybill J.R., Sugar A.M., Catanzaro A. (1994). NIAID mycoses study group multicenter trial of oral itraconazole therapy for invasive aspergillosis. Am. J. Med..

[50] Lamy T., Bernard M., Courtois A., Jacquelinet C., Chevrier S., Dauriac C., Grulois I., Guiguen C., Le Prise P. (1998). Prophylactic use of itraconazole for the prevention of invasive pulmonary aspergillosis in high risk neutropenic patients. Leuk. Lymphoma.

[51] Ghannoum M., Long L., Larkin E.L., Isham N., Sherif R., Borroto-Esoda K., Barat S., Angulo D. (2018). Evaluation of the antifungal activity of the novel oral glucan synthase inhibitor SCY-078, singly and in combination, for the treatment of invasive aspergillosis. Antimicrob. Agents Chemother..

[52] Katragkou A., McCarthy M., Meletiadis J., Petraitis V., Moradi P.W., Strauss G.E., Fouant M.M., Kovanda L.L., Petraitiene R., Roilides E. (2014). In vitro combination of isavuconazole with micafungin or amphotericin B deoxycholate against medically important molds. Antimicrob. Agents Chemother..

[53] Elefanti A., Mouton J.W., Verweij P.E., Tsakris A., Zerva L., Meletiadis J. (2013). Amphotericin B- and voriconazole-echinocandin combinations against *Aspergillus* spp.: Effect of serum on inhibitory and fungicidal interactions. Antimicrob. Agents Chemother..

[54] Seyedmousavi S., Meletiadis J., Melchers W.J.G., Rijs A.J.M.M., Mouton J.W., Verweij P.E. (2012). In vitro interaction of voriconazole and anidulafungin against triazole-resistant *Aspergillus fumigatus*. Antimicrob. Agents Chemother..

[55] Seyedmousavi S., Brüggemann R.J.M., Melchers W.J.G., Rijs A.J.M.M., Verweij P.E., Mouton J.W. (2012). Efficacy and pharmacodynamics of voriconazole combined with anidulafungin in azole-resistant invasive aspergillosis. J. Antimicrob. Chemother..

[56] Caillot D., Bassaris H., McGeer A., Arthur C., Prentice H.G., Seifert W., De Beule K. (2001). Intravenous itraconazole followed by oral itraconazole in the treatment of invasive pulmonary aspergillosis in patients with hematologic malignancies, chronic granulomatous disease, or AIDS. Clin. Infect. Dis..

[57] Lee J.W., Amantea M.A., Francis P.A., Navarro E.E., Bacher J., Pizzo P.A., Walsh T.J. (1994). Pharmacokinetics and safety of a unilamellar liposomal formulation of amphotericin B (AmBisome) in rabbits. Antimicrob. Agents Chemother..

[58] Walsh T.J., Lutsar I., Driscoll T., Dupont B., Roden M., Ghahramani P., Hodges M., Groll A.H., Perfect J.R. (2002). Voriconazole in the treatment of aspergillosis, scedosporiosis and other invasive fungal infections in children. Pediatr. Infect. Dis. J..

[59] Walsh T.J., Pappas P., Winston D.J., Lazarus H.M., Petersen F., Raffalli J., Yanovich S., Stiff P.J., Greenberg R., Donowitz G. (2002). Voriconazole compared with liposomal amphotericin B for empirical antifungal therapy in patients with neutropenia and persistent fever. N. Engl. J. Med..

[60] Cornely O.A., Maertens J., Winston D.J., Perfect J., Ullmann A.J., Walsh T.J., Helfgott D., Holowiecki J., Stockelberg D., Goh Y.-T. (2007). Posaconazole vs. fluconazole or itraconazole prophylaxis in patients with neutropenia. N. Engl. J. Med..

[61] Herbrecht R., Denning D.W., Patterson T.F., Bennett J.E., Greene R.E., Oestmann J.-W., Kern W.V., Marr K.A., Ribaud P., Lortholary O. (2002). Voriconazole versus amphotericin B for primary therapy of invasive aspergillosis. N. Engl. J. Med..

[62] Petraitiene R., Petraitis V., Lyman C.A., Groll A.H., Mickiene D., Peter J., Bacher J., Roussillon K., Hemmings M., Armstrong D. (2004). Efficacy, safety, and plasma pharmacokinetics of escalating dosages of intravenously administered ravuconazole lysine phosphoester for treatment of experimental pulmonary aspergillosis in persistently neutropenic rabbits. Antimicrob. Agents Chemother..

[63] Groll A.H., Walsh T.J. (2006). Antifungal efficacy and pharmacodynamics of posaconazole in experimental models of invasive fungal infections. Mycoses.

[64] Walsh T.J., Raad I., Patterson T.F., Chandrasekar P., Donowitz G.R., Graybill R., Greene R., Hachem R., Hadley S., Herbrecht R. (2007). Treatment of invasive aspergillosis with posaconazole in patients who are refractory to or intolerant of conventional therapy: An externally controlled trial. Clin. Infect. Dis..

[65] Kontoyiannis D.P., Selleslag D., Mullane K., Cornely O.A., Hope W., Lortholary O., Croos-Dabrera R., Lademacher C., Engelhardt M., Patterson T.F. (2017). Impact of unresolved neutropenia in patients with neutropenia and invasive aspergillosis: A post hoc analysis of the SECURE trial. J. Antimicrob. Chemother..

[66] Krel M., Petraitis V., Petraitiene R., Jain M.R., Zhao Y., Li H., Walsh T.J., Perlin D.S. (2014). Host biomarkers of invasive pulmonary aspergillosis to monitor therapeutic response. Antimicrob. Agents Chemother..

[67] Kovanda L.L., Petraitiene R., Petraitis V., Walsh T.J., Desai A., Bonate P., Hope W. (2016). Pharmacodynamics of isavuconazole in experimental invasive pulmonary aspergillosis: Implications for clinical breakpoints. J. Antimicrob. Chemother..

[68] Petraitis V., Petraitiene R., Moradi P.W., Strauss G.E., Katragkou A., Kovanda L.L., Hope W., Walsh T.J. (2016). Pharmacokinetics and concentration-dependent efficacy of isavuconazole for treatment of experimental invasive pulmonary aspergillosis. Antimicrob. Agents Chemother..

[69] Petraitis V., Petraitiene R., Katragkou A., Maung B.B.W., Naing E., Kavaliauskas P., Barat S., Borroto-Esoda K., Azie N., Angulo D. (2020). Combination therapy with ibrexafungerp (formerly SCY-078), a first-in-class triterpenoid inhibitor of (1->3)-β-D-glucan synthesis, and isavuconazole for treatment of experimental invasive pulmonary aspergillosis. Antimicrob. Agents Chemother..

[70] Petraitis V., Petraitiene R., Hope W., Walsh T.J. (2017). Endpoint assessment in rabbit models of invasive pulmonary aspergillosis and mucormycosis. Methods Mol. Biol..

[71] Al-Nakeeb Z., Petraitis V., Goodwin J., Petraitiene R., Walsh T.J., Hope W. (2015). Pharmacodynamics of amphotericin B deoxycholate, amphotericin B lipid complex, and liposomal amphotericin B against *Aspergillus fumigatus*. Antimicrob. Agents Chemother..

[72] Francesconi A., Kasai M., Petraitiene R., Petraitis V., Kelaher A.M., Schaufele R., Hope W., Shea Y.R., Bacher J., Walsh T.J. (2006). Characterization and comparison of galactomannan enzyme immunoassay and quantitative real-time PCR assay for detection of *Aspergillus fumigatus* in bronchoalveolar lavage fluid from experimental invasive pulmonary aspergillosis. J. Clin. Microbiol..

[73] Walsh T.J., Wissel M.C., Grantham K.J., Petraitiene R., Petraitis V., Kasai M., Francesconi A., Cotton M.P., Hughes J.E., Greene L. (2011). Molecular detection and species-specific identification of medically important *Aspergillus* species by real-time PCR in experimental invasive pulmonary aspergillosis. J. Clin. Microbiol..

[74] Gonzales D.A., De Torre-Minguela C., Wang H., Devor C.B., Munson P.J., Ying S.-X., Kern S.J., Petraitiene R., Levens D.L., Walsh T.J. (2010). Protein expression profiles distinguish between experimental invasive pulmonary aspergillosis and *Pseudomonas* pneumonia. Proteomics.

[75] Walsh T.J., Petraitis V., Petraitiene R., Field-Ridley A., Sutton D., Ghannoum M., Sein T., Schaufele R., Peter J., Bacher J. (2003). Experimental pulmonary aspergillosis due to *Aspergillus terreus*: Pathogenesis and treatment of an emerging fungal pathogen resistant to amphotericin B. J. Infect. Dis..

[76] Roberts J., Schock K., Marino S., Andriole V.T. (2000). Efficacies of two new antifungal agents, the triazole ravuconazole and the echinocandin LY-303366, in an experimental model of invasive aspergillosis. Antimicrob. Agents Chemother..

[77] Jeans A.R., Howard S.J., Al-Nakeeb Z., Goodwin J., Gregson L., Warn P.A., Hope W. (2012). Combination of voriconazole and anidulafungin for treatment of triazole-resistant *Aspergillus fumigatus* in an in vitro model of invasive pulmonary aspergillosis. Antimicrob. Agents Chemother..

[78] Petraitis V., Petraitiene R., McCarthy M.W., Kovanda L.L., Zaw M.H., Hussain K., Shaikh N., Maung B.B.W., Sekhon N.K., Hope W.W. (2017). Combination therapy with isavuconazole and micafungin for treatment of experimental invasive pulmonary aspergillosis. Antimicrob. Agents Chemother..

[79] Stevens V.M., Mueller S.W., Reynolds P.M., MacLaren R., Kiser T.H. (2020). Extrapolating antifungal animal data to humans—Is it reliable?. Curr. Fungal. Infect. Rep..

[80] Siopi M., Siafakas N., Vourli S., Mouton J.W., Zerva L., Meletiadis J. (2016). Dose optimization of voriconazole/anidulafungin combination against *Aspergillus fumigatus* using an in vitro pharmacokinetic/pharmacodynamic model and response surface analysis: Clinical implications for azole-resistant aspergillosis. J. Antimicrob. Chemother..

[81] O’Shaughnessy E.M., Meletiadis J., Stergiopoulou T., Demchok J.P., Walsh T.J. (2006). Antifungal interactions within the triple combination of amphotericin B, caspofungin and voriconazole against *Aspergillus* species. J. Antimicrob. Chemother..

[82] Marr K.A., Schlamm H., Herbrecht R., Rottinghaus S.T., Bow E.J., Cornely O.A., Heinz W.J., Jagannatha S., Koh L.P., Kontoyiannis D.P. (2015). Combination antifungal therapy for invasive aspergillosis. Ann. Intern. Med..

[83] Berenguer J., Ali N.M., Allende M.C., Lee J., Garrett K., Battaglia S., Piscitelli S.C., Rinaldi M.G., Pizzo P.A., Walsh T.J. (1994). Itraconazole for experimental pulmonary aspergillosis: Comparison with amphotericin B, interaction with cyclosporin A, and correlation between therapeutic response and itraconazole concentrations in plasma. Antimicrob. Agents Chemother..

[84] Berenguer J., Allende M.C., Lee J.W., Garrett K., Lyman C., Ali N.M., Bacher J., Pizzo P.A., Walsh T.J. (1995). Pathogenesis of pulmonary aspergillosis. Granulocytopenia versus cyclosporine and methylprednisolone-induced immunosuppression. Am. J. Respir. Crit. Care Med..

